# A meta-analysis comparing the performance of narrowband CE-Chirp and 500 Hz tone burst stimuli in recording cervical vestibular evoked myogenic potential (cVEMP)

**DOI:** 10.1038/s41598-024-64402-z

**Published:** 2024-06-26

**Authors:** Mohd Normani Zakaria, Athar Mazen Rasmi Abdallatif, Wan Najibah Wan Mohamad, Mohd Fadzil Nor Rashid, Robert Patuzzi, Baharudin Abdullah, Rosdan Salim, Marniza Omar

**Affiliations:** 1 Audiology Programme, School of Health Sciences, Universiti Sains Malaysia, Kubang Kerian, Kelantan Malaysia; 2 Department of Otorhinolaryngology, School of Medical Sciences, Universiti Sains Malaysia, 16150 Kubang Kerian, Kelantan Malaysia; 3 Centre for Rehabilitation and Special Needs Studies, Faculty of Health Sciences, Universiti Kebangsaan Malaysia, Kuala Lumpur, Malaysia

**Keywords:** Medical research, Neurology

## Abstract

Due to contradictory outcomes in the literature, the aim of this meta-analysis is to verify whether the narrowband (NB) CE-Chirp stimulus (centred at 500 Hz) would produce more robust cervical vestibular evoked myogenic potential (cVEMP) responses relative to the conventional 500 Hz tone burst. The literature search was conducted using PubMed, Scopus, and Web of Science databases and the terms used were “vestibular evoked myogenic potential” and “chirp”. The cVEMP parameters to be analysed were P1 latency, N1 latency, and P1–N1 amplitude. A total of 59 potential articles were obtained from the database search. Eventually, five articles were found to be eligible for the meta-analysis (with *n* = 222). As found, P1 and N1 latencies of cVEMP were significantly shorter for the chirp stimulus (*p* < 0.001), with substantially large effect sizes. On the other hand, P1–N1 amplitude values were found to be not statistically different between the two stimuli (*p* = 0.189), with a small effect size. It appears that there is no indication to support the superiority of the NB CE-Chirp stimulus (centred at 500 Hz) in the cVEMP testing (relative to the conventional 500 Hz tone burst). In particular, both stimuli produce comparable P1–N1 amplitude values. Even though P1 and N1 latencies are statistically shorter for the chirp stimulus, this may not reflect that it should be the preferred stimulus for recording cVEMP responses (and the reasons for this are discussed accordingly).

## Introduction

Apart from having a prominent role in maintaining body posture, the otolith organs (i.e., saccule and utricle) are also responsive to sounds^1–4^. In view of this, if loud sounds are presented (and by placing several electrodes at specific locations), the status of the saccule (and its central connections) can be conveniently assessed^3,4^. Furthermore, to ensure the responses are robustly generated, an individual is asked to turn his/her head away from the test ear (where sounds are delivered) to sufficiently contract the sternocleidomastoid (SCM) muscle (i.e., the end portion of the saccular projections). This electrophysiological procedure is known as the cervical vestibular evoked myogenic potential (cVEMP)^3^. It consists of two prominent peaks, i.e., P1 (or P13) and N1 (or N23), which have been shown to be vestibular (rather than cochlea) in origin^3–6^. The cVEMP assessment has been studied in various peripheral and central disorders, including Meniere’s disease, superior canal dehiscence, vestibular neuritis, multiple sclerosis, brainstem lesions, etc., and the study outcomes are promising^3,4^. Due to its simplicity and non-invasiveness, it has been commonly used as part of the vestibular test battery in clinical settings^3,4^. On the other hand, another variation of VEMP, known as the ocular VEMP (oVEMP), is useful to assess the function of the utricle and superior vestibular nerve^2,4^.

In the field of electrophysiology, robust waveforms are characterized by larger amplitudes and shorter latencies^7^. In the earlier studies, clicks were the stimuli of interest in eliciting cVEMP waveforms^6,8,9^. Over the years, the low frequency tone bursts have been consistently found to produce larger cVEMP amplitudes and higher response rates^10–13^. As such, the low frequency tone such as a 500 Hz burst has been the stimulus of choice for clinical and research applications^3,4,13–15^. In recent years, there has been a surge of interest in researching the usefulness of chirp stimuli in the cVEMP testing^16–26^. It is worth mentioning that the chirp stimuli were specifically developed with the aim of producing enhanced amplitudes of auditory evoked potentials (AEPs), such as the auditory brainstem response (ABR) and the auditory steady state response (ASSR)^27–30^. Having more robust electrophysiological waveforms offers several benefits when assessing patients in clinical settings, such as more reliable results and shortened testing time (due to reduced sweeps)^7^.

Even though there are many types of chirp stimuli^17–21^, a narrowband (NB) CE-Chirp stimulus (frequencies from 360 to 720 Hz and centred at 500 Hz) has been commonly employed in the cVEMP studies^18,20,22–26^. The purpose of applying this chirp stimulus, which is commercially accessible in many types of AEP devices, is to ascertain whether it would produce more robust cVEMP responses than the conventional 500 Hz tone burst. If this is proven to be true, the NB CE-Chirp stimulus could be considered a more optimal stimulus (than the 500 Hz tone burst) in clinical practice. Nevertheless, contradictory outcomes have been reported in the literature. While the majority of studies found P1 and N1 latencies to be statistically shorter for the chirp stimulus^17,18,20–26^, inconsistent findings were reported regarding P1–N1 amplitude. In particular, relative to the 500 Hz tone burst, several studies found the P1–N1 amplitude to be larger for the chirp stimulus^18,20,22,23^. On the contrary, in other studies, the P1–N1 amplitude was found to be comparable between the two stimuli^24–26^, as well as lower for the chirp stimulus^17^. Owing to the conflicting findings in the literature, the aim of this meta-analysis is to provide more plausible evidence as to whether the NB CE-Chirp stimulus is superior to the conventional 500 Hz tone burst in the cVEMP testing.

## Methods

### Literature search strategy

This meta-analysis was registered in PROSPERO (CRD42024543110) and followed the guideline provided by the Preferred Reporting Items for Systematic reviews and Meta-Analyses (PRISMA) 2020 statement^31^. Three major databases were used (i.e., PubMed, Scopus, and Web of Science) and the literature search for the potential articles ended on March 31, 2023. Since the chirp-evoked cVEMP studies started in 2014, it was anticipated that the number of articles would not be as high as cVEMP studies employing clicks and/or tone bursts. As such, for the article search, the keywords used were “vestibular evoked myogenic potential” and “chirp”.

We aimed to include studies based on the PICO framework. In particular, the population (P) is a group of healthy adults, the intervention (I) or exposure is the assessment of interest (i.e., air conduction cVEMP), the comparison (C) is about comparing the performance of the commercially available NB CE-Chirp (centred at 500 Hz) and 500 Hz tone burst stimuli, and the outcomes (O) are the cVEMP results (sample size, mean and standard deviation values for P1 latency, N1 latency, and P1–N1 amplitude). Studies that recorded oVEMP, used methods other than the air conduction to record cVEMP (e.g., bone conduction, galvanic, etc.), included pathological participants (e.g., those with vestibular disorders or hearing impairment), and used other types of chirp stimuli (e.g., custom-built chirp stimuli) were excluded.

For the initial search, all types of studies were considered. Duplicates were identified and excluded accordingly. Subsequently, the articles were identified based on the inclusion and exclusion criteria (by evaluating the title and abstract words, indexed terms, and author keywords). Studies reported as editorials, letters, case reports, meta-analyses, and reviews, were excluded. Additional articles were searched based on the reference lists of the listed papers (i.e., a citation search method). The selection of studies and data extraction were conducted by two authors, and any disagreements were resolved through discussions. Three authors were involved (and blinded to each other's decisions) in evaluating the methodological quality of the shortlisted studies, and the Joanna Briggs Institute (JBI)’s critical appraisal checklist (for cross-sectional studies) was used for this purpose^32^. In this checklist, there are eight questions to be addressed (and the options are “Yes”, “No”, “Unclear” and “Not applicable”), and the evaluators must also provide the overall appraisal (whether to choose “Include”, “Exclude” or “Seek further info”). The methodological quality was set at a 50% cut-off, in which any study that scored below 50% would be excluded^33^.

### Data analysis

The meta-analysis for comparing cVEMP results (P1 latency, N1 latency, and P1–N1 amplitude) between the NB CE-Chirp (centred at 500 Hz) and 500 Hz tone burst stimuli was performed using the MedCalc software (version 20.006, Ostend, Belgium). The continuous data (i.e., sample size, mean, and standard deviation values for both stimuli) from the selected studies were computed to measure the standardized mean difference (SMD). Calculated based on the Hedges’ *g* effect size, an SMD of 0.2 indicates a small effect, an SMD of 0.5 implies a medium effect, and an SMD of 0.8 represents a large effect^34^. Additionally, the SMD value was considered statistically significant (i.e., *p* < 0.05) when the value 0 was not within the 95% confidence interval (CI). As recommended, if the Cochran’s *Q* test was significant (*p* < 0.05) and *I*^*2*^ statistic > 50% (suggestive of the presence of substantial heterogeneity), the random effects model was used to describe the data^35,36^. Otherwise, the fixed effects model was chosen (if the data were not considerably heterogeneous) for the data analysis. The Egger's test and Begg’s rank test were used to assess any potential publication bias^37,38^. Prior to the meta-analysis, the Fleiss’ kappa analysis was employed to measure the agreement between the three evaluators (for the methodological quality assessment of the shortlisted articles). Herein, kappa values of < 0.20, 0.21–0.40, 0.41–0.60, 0.61–0.80 and 0.81–1.00 indicate poor, fair, moderate, good and very good agreements, respectively^39^.

## Results

### Literature search results

**Figure 1:**
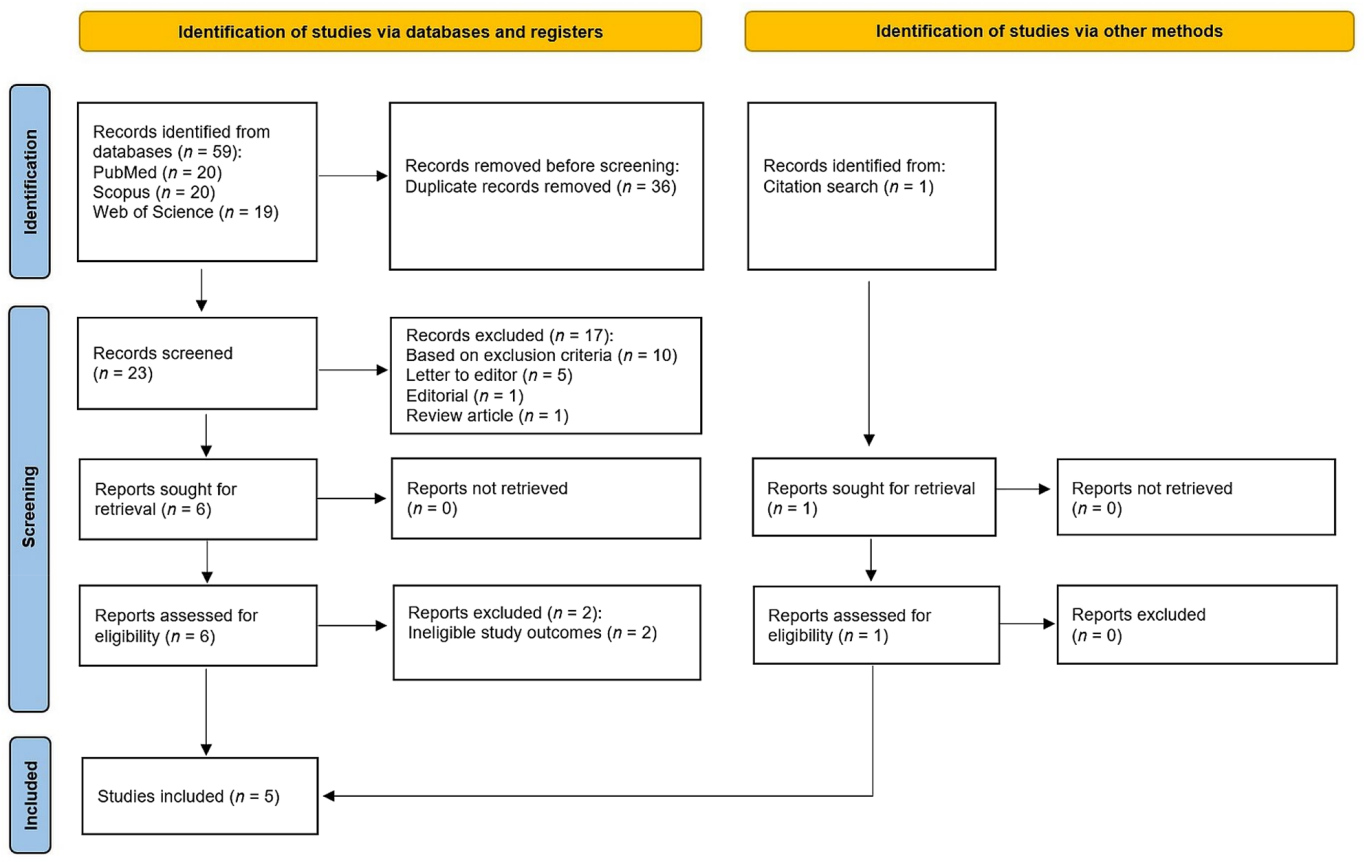
The flowchart of the article selection for the meta-analysis.

Figure 1 illustrates the selection process of the articles categorised under “identification”, “screening” and “included”. In the identification section, the search results using the three databases revealed 59 potential articles. Of this, 36 studies were duplicates and removed. In the screening section, of 23 articles, 17 were excluded as they were published as letters to the editor (*n* = 5), editorials (*n* = 1), or review articles (*n* = 1) and did not fulfil the inclusion criteria (*n* = 10). In particular, those did not satisfy the inclusion criteria as they were oVEMP studies (*n* = 4), recorded masseteric VEMP (mVEMP) (*n* = 1), employed broadband chirp stimuli (*n* = 2), used custom-built chirp stimuli (*n* = 2), and did not compare cVEMP results between the two stimuli of interest (*n* = 1). The full texts of the remaining six articles were reviewed accordingly to determine their eligibility. Subsequently, two articles were excluded due to ineligible study outcomes. In particular, studies conducted by Mat et al.^22^ and Neupane and Lodha^26^ did not provide the respective mean and standard deviation values of cVEMP parameters. Additionally, an article authored by Moinudeen et al.^20^ was retrieved using the citation search method (Fig. 1), and it was then found to be eligible. Finally, five articles were shortlisted to be included in the meta-analysis. In terms of the methodological quality assessment, the three evaluators consensually agreed to include all five articles. It is worth mentioning that all the articles satisfied the minimum requirement to be included (average scores ranging from 75.0 to 100.0%, and none of them had scores less than 50.0%). Moreover, based on the Fleiss’ kappa values (*k* = 0.73–1.00), the agreement between the evaluators ranged from good to very good. Taken together, all articles included in this meta-analysis were considered to be of good quality, and the desired statistical results could be achieved.

### Profiles of the included studies

Table 1 shows the profiles of the studies included in this meta-analysis. As revealed, the chosen studies were those carried out by Wang et al.^18^, Moinudeen et al.^20^, Ocal et al.^24^, Reddy et al.^23^, and Aydin and Erbek^25^, contributing to a total of 222 participants. Four out of five studies recorded cVEMP responses from healthy young adults (with comparable ages)^18,20,23,24^. In the study by Aydin and Erbek^25^, cVEMP and oVEMP results produced by NB CE-Chirp and 500 Hz tone burst stimuli were compared between participants with acute peripheral vestibular pathologies (*n* = 50, mean age = 53.2 ± 11.9 years) and healthy controls (*n* = 54). Herein, they only stated that the gender and age of the healthy participants were matched to those of the participants with vestibular pathologies (without specifying the mean age)^25^. Three studies used Interacoustics Eclipse to record cVEMP responses^23–25^. Even though the other two studies utilized different devices (i.e., GSI Audera Pro and Neurosoft Neuro-Audio)^18,20^, all of them compared the performance of the commercially available NB CE-Chirp stimulus (with frequencies from 360 to 720 Hz and centred at 500 Hz) with the 500 Hz tone burst (with essentially similar settings, i.e., 6 ms duration). Four out of five studies preferred to use 100 dB nHL as the intensity level to record cVEMP waveforms^18,20,24,25^. Additionally, all studies employed similar placement of the electrodes, with the non-inverting electrode placed at the midpoint of the SCM muscle, the negative electrode on the sternum, and the ground electrode on the forehead. The use of visual feedback to control for the desired SCM contraction was reported in all studies. In particular, three studies stated that the rectified electromyography (EMG) level was maintained to be at least 50 µV during the cVEMP testing^18,23,25^. On the other hand, the lower limit of the SCM contraction was set at 20 µV in the study by Ocal et al.^24^, while Moinudeen et al.^20^ did not specify the minimum level of rectified EMG. Collectively, the profiles of the chosen studies were essentially similar and suitable for the meta-analysis.

**Table 1:** The profiles of studies included in the meta-analysis.

Authors	Year	*n*	Mean age (years)	Sex (male/female)	Device	Tone burst setting	Stimulus level
Wang et al.^18^	2014	30	24	9/21	Audera (GSI)	Rarefaction, 5 ms (“2–1–2”)	100 dB nHL
Moinudeen et al.^20^	2020	30	22	15/15	Neuro-Audio (Neurosoft)	Rarefaction, 6 ms (“2–2–2”)	100 dB nHL
Ocal et al.^24^	2021	50	26.7	21/29	Eclipse (Interacoustics)	6 ms (“2–2–2”)	100 dB nHL
Reddy et al.^23^	2022	58	25	20/38	Eclipse (Interacoustics)	Rarefaction, 6 ms (“2–2–2”)	95 dB nHL
Aydin and Erbek^25^	2023	54	Not stated	Not stated	Eclipse (Interacoustics)	Rarefaction, 6 ms (“2–2–2”)	100 dB nHL

### Analysis of response rate

Of five studies, four of them reported the response rate of cVEMP for both stimuli. As shown in Table 2, the cVEMP response rate ranged from 98 to 100% for the NB CE-Chirp stimulus. For the 500 Hz tone burst, the response rate was between 91 and 100%. It is worth stating that a response rate of 100% for both stimuli was seen in studies conducted by Wang et al.^18^ and Ocal et al.^24^, which used an intensity level of 100 dB nHL for eliciting cVEMP responses.

**Table 2:** The response rate of cervical vestibular evoked myogenic potential (cVEMP) elicited by the narrowband (NB) CE-Chirp (centred at 500 Hz) and 500 Hz tone burst stimuli for the studies included in the meta-analysis.

Authors	*n*	Response rate (%)	
		NB CE-Chirp	500 Hz tone burst
Wang et al.^18^	30	100	100
Moinudeen et al.^20^	30	Not stated	Not stated
Ocal et al.^24^	50	100	100
Reddy et al.^23^	58	100	91
Aydin and Erbek^25^	54	98	97

### Analysis of P1 latency

**Figure 2:**
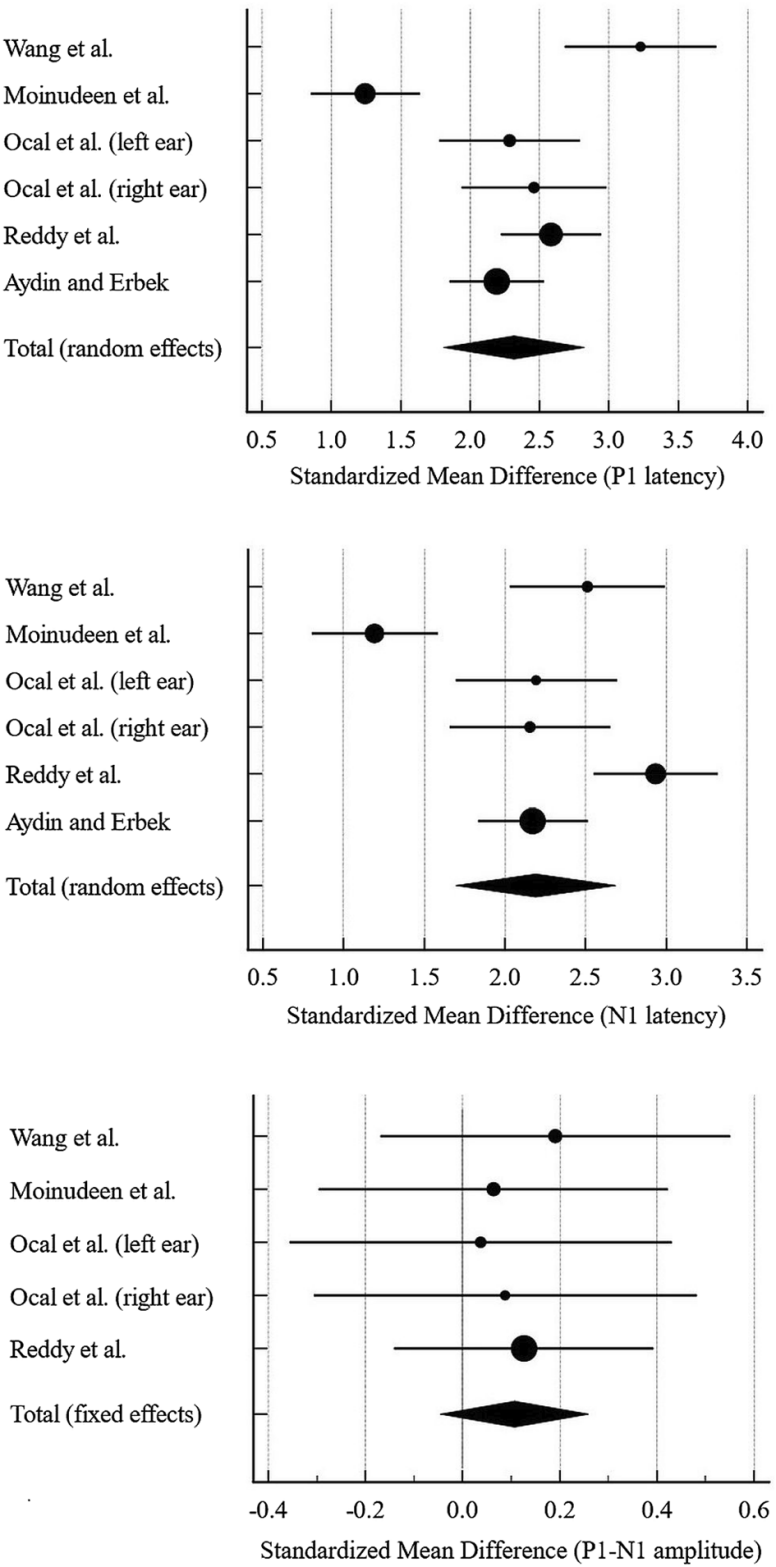
The forest plot of the meta-analysis when comparing narrow band (NB) CE-Chirp and 500 Hz tone burst stimuli for P1 latency (upper plot), N1 latency (middle plot) and P1–N1 amplitude (lower plot).

The forest plot of P1 latency (showing SMD) is illustrated in Fig. 2. It is worth stating that four out of five studies provided cVEMP data by combining left and right ear findings^18,20,23,25^. However, in the study by Ocal et al.^24^, the pooled left and right data were not provided. As such, in this meta-analysis, the left and right data provided by Ocal et al.’s study were analysed separately. Since substantial heterogeneity was observed (Cochran’s *Q* test of *p* < 0.001 and *I*^*2*^ statistic of 88.1%), the random effects model was chosen to compare the P1 latency data between the two stimuli. Of note, no evidence of publication bias was observed based on the Egger’s test (*p* = 0.401) and the Begg’s test (*p* = 0.189). Table 3 shows the mean, standard deviation, and SMD values of P1 latency for each study. As revealed, the individual SMD values ranged from 1.25 to 3.23, indicating notably large effect sizes. By combining the data (with *n* = 434 ears and *n* = 422 ears for NB CE-Chirp and 500 Hz tone burst stimuli, respectively), the total SMD value was also high, i.e., 2.32 (95% CI 1.81–2.83), and statistically significant (*p* < 0.001). This analysis ascertains that the P1 latency of cVEMP is statistically shorter for the chirp stimulus, and the difference between the stimuli is substantially large.

**Table 3:** Mean and standard deviation (SD) values of P1 latency for narrowband (NB) CE-Chirp and 500 Hz tone burst stimuli for the studies included in the meta-analysis.

Authors	NB CE-Chirp		500 Hz tone burst		SMD (95% CI)
	*n* (ears)	Mean ± SD (ms)	*n* (ears)	Mean ± SD (ms)	
Wang et al.^18^	60	4.91 ± 2.11	60	11.81 ± 2.14	3.23 (2.68–3.77)
Moinudeen et al.^20^	60	12.61 ± 3.06	60	16.40 ± 2.99	1.25 (0.85–1.64)
Ocal et al.^24^ (left ear)	50	10.36 ± 2.39	50	16.00 ± 2.51	2.28 (1.78–2.79)
Ocal et al.^24^ (right ear)	50	10.56 ± 2.27	50	16.08 ± 2.18	2.46 (1.94–2.99)
Reddy et al.^23^	108	11.12 ± 2.80	98	18.53 ± 2.92	2.58 (2.21–2.96)
Aydin and Erbek^25^	106	12.42 ± 1.60	104	16.05 ± 1.70	2.19 (1.85–2.54)
Total	434		422		2.32 (1.81–2.83)
*P* value					< 0.001

Standardized mean difference (SMD) and the total SMD (with the respective* p* value) are shown (based on the random effects model).

### Analysis of N1 latency

The meta-analysis of the N1 latency of cVEMP is shown in Fig. 2 and Table 4. Overall, a similar pattern was observed, i.e., the difference in the N1 latency between the NB CE-Chirp and 500 Hz tone burst stimuli was substantially large, with effect sizes ranging from 1.19 to 2.95 across the studies (Table 4). By means of the random effects model, the total SMD value was 2.19 (95% CI 1.70–2.69) and statistically significant (*p* < 0.001). These results verify the notion that the N1 latency for the chirp stimulus is significantly and substantially shorter (relative to the 500 Hz tone burst). The random effects method was applied as the data revealed substantial heterogeneity (Cochran’s *Q* test of *p* < 0.001 and *I*^*2*^ statistic of 87.9%). Based on the results of Egger’s test (*p* = 0.772) and Begg’s test (*p* = 0.851), no publication bias was noted.

**Table 4:** Mean and standard deviation (SD) values of N1 latency for narrowband (NB) CE-Chirp and 500 Hz tone burst stimuli for the studies included in the meta-analysis.

Authors	NB CE-Chirp		500 Hz tone burst		SMD (95% CI)
	*n* (ears)	Mean ± SD (ms)	*n* (ears)	Mean ± SD (ms)	
Wang et al.^18^	60	11.88 ± 2.78	60	19.10 ± 2.93	2.51 (2.03–2.99)
Moinudeen et al.^20^	60	18.71 ± 2.78	60	22.36 ± 3.28	1.19 (0.80–1.58)
Ocal et al.^24^ (left ear)	50	19.06 ± 2.30	50	24.70 ± 2.78	2.19 (1.69–2.69)
Ocal et al.^24^ (right ear)	50	19.36 ± 2.70	50	25.30 ± 2.77	2.16 (1.66–2.65)
Reddy et al.^23^	108	17.52 ± 2.54	98	25.98 ± 3.17	2.95 (2.55–3.35)
Aydin and Erbek^25^	106	21.56 ± 1.90	104	25.93 ± 2.10	2.18 (1.83–2.52)
Total	434		422		2.19 (1.70–2.69)
*P* value					< 0.001

Standardized mean difference (SMD) and the total SMD (with the respective* p* value) are shown (based on the random effects model).

### Analysis of P1–N1 amplitude

Table 5 shows the descriptive data and SMD values of the P1–N1 amplitude for the chosen studies. The forest plot of the P1–N1 amplitude based on the meta-analysis is depicted in Fig. 2. Of note, since the study by Aydin and Erbek did not provide the respective standard deviation data^25^, this study was not included. Therefore, the meta-analysis of the P1–N1 amplitude was carried out with *n* = 328 ears (for the NB CE-Chirp stimulus) and *n* = 318 ears (for the 500 Hz tone burst). As revealed in Table 5, unlike the results of P1 and N1 latencies, the individual SMD values of the P1–N1 amplitude revealed small effect sizes (ranging from 0.04 to 0.19). Since there was no evidence that the combined data were heterogeneous (Cochran’s* Q* test of *p* = 0.978 and *I*^*2*^ statistic of 0.0%), the fixed effects model was employed. Subsequently, the total SMD value was found to be notably low, i.e., 0.10 (95% CI − 0.05 to 0.26), and not statistically significant (*p* = 0.189). These results provide plausible evidence that both stimuli elicit cVEMP responses with equivalent P1–N1 amplitude values. It is worth stating that there was no evidence of publication bias when analysing the P1–N1 amplitude data, with *p* = 0.443 (for the Egger’s test) and *p* = 0.624 (for the Begg’s test).

**Table 5:** Mean and standard deviation (SD) values of P1–N1 amplitude for narrowband (NB) CE-Chirp and 500 Hz tone burst stimuli for the studies included in the meta-analysis.

Authors	NB CE-Chirp		500 Hz tone burst		SMD (95% CI)
	*n* (ears)	Mean ± SD (µV)	*n* (ears)	Mean ± SD (µV)	
Wang et al.^18^	60	14.42 ± 5.50	60	13.33 ± 5.85	0.19 (− 0.17–0.55)
Moinudeen et al.^20^	60	70.15 ± 25.45	60	68.45 ± 28.11	0.06 (− 0.30–0.42)
Ocal et al.^24^ (left ear)	50	52.90 ± 24.49	50	51.95 ± 25.47	0.04 (− 0.36–0.43)
Ocal et al.^24^ (right ear)	50	56.99 ± 27.07	50	55.21 ± 28.54	0.06 (− 0.33–0.46)
Reddy et al.y^23^	108	74.99 ± 35.87	98	70.46 ± 35.60	0.13 (− 0.15–0.40)
Total	328		318		0.10 (− 0.05–0.26)
*P* value					0.189

Standardized mean difference (SMD) and the total SMD (with the respective* p* value) are shown (based on the fixed effects model).

## Discussion

Since vestibular disorders are common among adults^40^, getting an accurate clinical diagnosis is undoubtedly imperative. Those with serious vestibular symptoms should receive prompt and appropriate treatments after being diagnosed. To aid in the appropriate clinical decisions, specific vestibular assessments are carried out, and the results are integrated accordingly. Before the emergence of cVEMP, there was no clinical assessment to measure the integrity of the saccule and inferior vestibular nerve in an objective manner^41^. Due to its simplicity (as it can be carried out using the existing AEP machines), non-invasiveness, and promising study outcomes, it is advantageous to have the cVEMP testing in clinical settings^3,4^.

Choosing the “right” stimulus is essential when recording cVEMP responses so that reliable results can be obtained in a shorter testing time. As previously mentioned, the low frequency tone burst (e.g., 500 Hz) has been recommended for the cVEMP testing for both clinical and research purposes^3,4,13–15^. Compared to the click stimuli, the respective tone bursts have been consistently found to produce cVEMP responses with significantly higher amplitudes but longer latencies^11–13^. Getting higher cVEMP amplitudes with the low frequency tone bursts is sensible given that the best frequencies of the saccule are in the range of 400–800 Hz^3,4,10,14,15^. Since the otolith organs (including the saccule) were found to be sensitive to changes in acceleration over time^42^, shorter cVEMP latencies with the click stimuli (with an abrupt onset) were rather expected.

In order to appreciate the strengths and limitations of a new stimulus (of interest) in recording cVEMP waveforms, getting the research outcomes from healthy participants is the first step. Furthermore, since vestibular disorders are more common in adults, studying this age group appears to be a sensible approach. Ultimately, it is of interest to see whether the statistically significant results are also indicative of clinically significant outcomes. In view of this, effect size measures are among the recommended statistical analyses to suggest whether the results are clinically significant^43–45^. In this regard, if the chirp stimulus is proven to be more optimal (from statistical and clinical perspectives), there would be a clear indication to “replace” the conventional 500 Hz tone burst with this “new” stimulus in the cVEMP testing. In ABR and ASSR studies, the superiority of the chirp stimuli has been well demonstrated^27–30,41^. Nevertheless, the “superiority” of the chirp stimuli in the cVEMP testing (relative to the low frequency tone bursts) is not yet justified as contradictory outcomes have been reported in the literature. In an effort to provide credible evidence regarding this matter, we performed the meta-analysis for studies that compared the cVEMP results (P1 latency, N1 latency, and P1–N1 amplitude) between the NB CE-Chirp (frequencies from 360 to 720 Hz and centred at 500 Hz) and 500 Hz tone burst stimuli among healthy adults. Of note, this type of chirp stimulus was chosen for several reasons. First, since the 500 Hz tone burst is the most common stimulus employed in the previous studies, using a comparable stimulus (such as the NB CE-Chirp with a centre frequency of 500 Hz) seems like a reasonable decision in comparative studies. Second, compared to other types of chirp stimuli (e.g., broadband chirps, custom-built chirps, etc.), the NB CE-Chirp has been the stimulus of interest in many studies (in this regard, performing the meta-analysis is possible as the number of studies is sufficient). Lastly, this chirp stimulus is commercially available in many types of AEP devices (i.e., if this stimulus is proven to be a better option, it can be conveniently utilised using the existing AEP devices available in clinics).

Generally, the response rates of cVEMP produced by the two stimuli were high (more than 90%). Of the four studies that reported the cVEMP response rates, three of them reported a 100% response rate for the NB CE-Chirp stimulus^18,23,24^. While for the 500 Hz tone burst, a 100% response rate was reported by two studies^18,24^. The lowest cVEMP response rate (91%) was noted for the 500 Hz tone burst^23^. In view of this, it can be concluded that while both stimuli produce high cVEMP response rates, the NB CE-Chirp stimulus performs slightly better than the 500 Hz tone burst. It is essential to note that the use of higher intensity levels would affect the cVEMP response rates^4,13^, and this trend can be seen in this analysis. Herein, of the four studies that employed an intensity level of 100 dB nHL for recording cVEMP responses, two of them produced a 100% response rate for both stimuli^18,24^. The lowest cVEMP response rate for the 500 Hz tone burst was indeed noted in the study that utilized a lower intensity level (i.e., 95 dB nHL)^23^.

As clearly shown, relative to the 500 Hz tone burst, the P1 and N1 latencies were found to be statistically shorter for the chirp stimulus. In accordance with this, the individual and total SMD values indicated that the differences in the cVEMP latencies between the two stimuli were substantially large. In view of this, if the chirp stimulus is to be used for clinical applications, specific normative data are needed. Several reasons were proposed as to why the cVEMP latencies were shorter when elicited by the chirp stimulus^17,18,22,23^. The first published study of a chirp-evoked cVEMP was conducted by Wang et al.^18^. They postulated that the shorter P1 and N1 latencies for the chirp stimulus were due to enhanced activation of the saccule. Specifically, since the chirp stimulus was designed to compensate for the cochlear traveling wave delay^27,28^, greater activation of the basilar membrane and enhanced neural synchrony would be observed when elicited by this stimulus. Subsequently, increased movement of endolymph was expected to occur, resulting in greater activation of the saccule^18^. Nevertheless, since the cochlea and saccule are distinctive anatomically and physiologically (and little is known regarding how the two stimuli are interpreted by the saccule), there is no evidence to support this postulation. Furthermore, if the enhanced activation was thought to occur in the saccule (when elicited by the chirp stimulus), there is no reason to explain why the cVEMP latencies became shorter (instead of getting larger cVEMP amplitudes). Several other studies proposed that the statistically longer P1 and N1 latencies for the 500 Hz tone burst (relative to the NB CE-Chirp stimulus) were due to the characteristic of neural firing^22,23,46^. In particular, the primary vestibular neurons may show double or triple firings in response to a single tone burst, and the prolonged cVEMP latencies may be due to the second or third spikes^46^. This viewpoint is reasonable if the comparison is made with the click stimulus (where the shorter cVEMP latencies could be due to its shorter duration). It is worth stating that the NB CE-Chirp (centred at 500 Hz) stimulus also has a long duration (i.e., 9 ms), and it seems possible for the “neural delay” to also occur. As such, the shorter cVEMP latencies for the chirp stimulus could not be justified by this notion. The “unique” chirp design is most likely the reason for getting cVEMP responses with shorter latencies when evoked by the chirp stimulus^17,23^. As reported elsewhere^27,30^, the commercially available chirp stimuli were designed in such a way that their onset was placed earlier than the onset of the respective tone burst stimuli (i.e., before 0 ms). In this respect, cVEMP waveforms with shorter latencies would “always” be expected when stimulated by the chirp stimuli^17^. In line with this notion, Walther and Cebulla compared their custom-built chirp stimulus (250–1000 Hz) with 500 Hz tone burst and click stimuli in eliciting cVEMP responses^19^. Interestingly, they found that the longest cVEMP latencies were produced by the custom-built chirp stimulus (with an unmodified onset)^19^. Until additional evidence is forthcoming, collectively, the shorter P1 and N1 latencies produced by the NB CE-Chirp stimulus are likely due to the temporal adjustment of the stimulus (as opposed to any physiological reason). This also implies that the shorter cVEMP latencies for the chirp stimulus do not necessarily represent the robustness of cVEMP responses (i.e., other indicators such as the P1–N1 amplitude should be considered).

It is rather interesting to note that even though three out of four studies revealed significantly larger P1–N1 amplitude values for the chirp stimulus^18,20,23^, the total SMD value obtained in this meta-analysis indicated a negligible and insignificant difference between the two stimuli. Moreover, the upper limit of the 95% CI of SMD was still in the range of a small effect size. This implies that the P1–N1 amplitude values produced by both stimuli are indeed comparable (and there is no supporting evidence that the chirp stimulus is more beneficial than the conventional 500 Hz tone burst in the cVEMP testing). If the chirp stimulus was hypothesised to be “superior” to the conventional 500 Hz tone burst, significant results would have been obtained in the meta-analysis (as the combined data had increased statistical power). It is worth noting that those studies did not conduct the effect size analysis when comparing the P1–N1 amplitude values between the NB CE-Chirp and 500 Hz tone burst stimuli (and perhaps if the effect size measure was included, they might have interpreted their results in a different way). The negligible difference in the cVEMP amplitude between the two stimuli can be justified. Since both stimuli contain the best stimulus frequency to record cVEMP responses (i.e., 500 Hz) and were presented at a similar intensity level, getting comparable amplitude values is therefore sensible. Even though the chirp stimulus comparatively has a “broader” frequency range (i.e., 360–720 Hz), it is anticipated that the inclusion of a few extra frequencies would not significantly alter the cVEMP responses. The status of the middle ear is another critical factor in the cVEMP testing^3,4^. In the absence of any middle ear pathology, it is possible for the cVEMP responses to be altered by the middle ear resonant frequency (depending on the stimulus frequency). Nevertheless, since the middle ear resonant frequency of healthy adults was reported to be in a range of 800–1200 Hz^41,47^, it is expected that this effect is negligible (which is supported by the comparable P1–N1 amplitude values produced by the two stimuli in this meta-analysis). In accordance with this, a study carried out by Jha et al. revealed that differences in the “best” frequency of cVEMP between young and old adults were not significantly attributed to the middle ear factor^48^.

Generally, stimuli presented at high intensity levels are recommended to produce cVEMP waveforms with higher response rates and larger amplitudes^4,13^. However, if the stimuli are excessively loud, adverse effects on cochlear function can be seen^49,50^. Of note, none of the studies included in this meta-analysis reported the equivalent sound energy exposure (L_Aeq_) in the cVEMP testing. Recall that four out of the five studies recorded cVEMP responses to chirp and tone burst stimuli presented at an intensity level of 100 dB nHL. In this regard, the sound energy exposure in reference to 85 dB L_Aeq_,_8 h_ or 105 dB L_Aeq,1 s_ should have been reported^4,49^.

This meta-analysis had several limitations. First, only five eligible articles were included, and perhaps better statistical outcomes would be obtained if more studies could be included. Second, we only analysed studies that provided raw or “uncorrected” amplitude data of cVEMP (as these data were commonly reported). In recent years, there has been a trend towards reporting “corrected” amplitude values in cVEMP studies^21,22,26^. Of note, the corrected amplitude approach is specifically beneficial when comparing cVEMP results between left and right ears^3,4^. In view of this, future meta-analysis studies may consider investigating the performance of the two stimuli of interest by means of their corrected P1–N1 amplitude values (when the number of studies is sufficient). Lastly, only studies that compared the performance of the commercially available NB CE-Chirp (centred at 500 Hz) and 500 Hz tone burst stimuli were included (based on the aforementioned rationales). Since the performance of other types of chirp stimuli appears to be promising as well^19,21^, studying the clinical usefulness of these stimuli can be the next step.

## Conclusion

When assessing healthy adults, it appears that there is no indication to support the superiority of the NB CE-Chirp stimulus (centred at 500 Hz) in the cVEMP testing (relative to the conventional 500 Hz tone burst). In particular, unlike chirp-evoked ABR findings, both stimuli produce comparable P1–N1 amplitude values. Even though P1 and N1 latencies are statistically shorter for the chirp stimulus, this may not imply that it produces more robust waveforms compared to the 500 Hz tone burst. Based on the SMD values, there is no clear advantage to incorporating the chirp stimulus into clinical practice (and the conventional 500 Hz tone burst is sufficient to serve this purpose). Nevertheless, further research comparing the sensitivity and specificity of these stimuli when assessing those with vestibular disorders can be advantageous (to gain better insight into which stimulus is the preferred option).

## Data Availability

The datasets used and/or analysed during the current study available from the corresponding author on reasonable request.
